# TRAINING IN HEALTH AND AGING POLICY MID CAREER—REFLECTING ON A POTENTIAL INFLECTION POINT

**DOI:** 10.1093/geroni/igad104.0025

**Published:** 2023-12-21

**Authors:** Benjamin Springgate

**Affiliations:** LSU Health - New Orleans, New Orleans, Louisiana, United States

## Abstract

The Health and Aging Policy Fellowship (HAPF) trains fellows to understand policymaking with the goal to improve the lives of older people across life domains. Comparatively few mid-career or more senior experts in healthcare or aging have direct experience with federal or state policymaking, or working to inform evidence-based health policy. As a senior physician faculty member at a School of Medicine and School of Public Health, in recent years the author has worked at state and local levels to inform evidence-based health policy. The HAPF offered an opportunity for the author to improve knowledge and understanding of decision-making and policymaking in Washington DC and at the federal level. Fellowship-sponsored training improved fellows’ understanding of health policy and potential effectiveness in federal health policy environments. Trainings through HAPF, the American Political Science Association, and Academy Health provided greater breadth and depth of knowledge on roles of various agencies, executive and congressional policymaking, and understanding factors influencing policymaking. This training enhanced the author’s readiness for fellowship activities in a U.S. Senate office. Exposure to experts in health and aging opened the door to fellows’ participation in efforts to improve opportunities for healthy aging and to advance age-friendly health systems. Work in the U.S. Senate includes exposure to federal, state, non-governmental stakeholders who can work together to influence policy to improve the lives of aging and older people. The author will highlight specific examples of training, policy opportunities, and policy challenges in the context of his fellowship.

